# Dr. Alder, on Quarantines, Contagion, and Fevers

**Published:** 1808-03

**Authors:** Thomas Alder

**Affiliations:** Edinburgh


### *To the Editors of the Medical and Physical Journal.*

GENTLEMEN,

IN my last, I took a view of the endemic and epidemic bad air in a cursory manner;—I might have dwelt much longer on this subject, for the sources of bad air are almost most innumerable, and the action of it upon the body is very extensive and morbiferous. I believe it would not be very difficult to prove that epidemic bad air has been as destructive as war and famine, particularly when assisted by the endemic or sporadic kind, or depressing passions:—it is astonishing, how clearly the most fatal epidemics can be traced to this cause; the African source of it alone, (which I have called the Æthiopian, as it comes from near Æthiopia, and the ancients in general speak of their plagues as coming from this quarter,) this air alone seems to have been, when assisted, as I have before noticed, more letheferous than all the kinds of contagion, among which, however, I admit none but those that are *specific* put together; and almost as fatal as the sword; which it would not be difficult to prove; and yet, bad as this African air is, and extensive in its range, we can scarcely ever find bad epidemics have prevailed, without the proofs that epidemic or endemic bad air has been about the same time copiously emitted from other sources; or formed from rain, or set at liberty by a thaw.

*Endemic air.* But besides this, the endemic and sporadic bad air have great effect; the endemic exists not only in the places which have been particularized, where it is abundant and very mortal, but in a less degree in many others: every marshy, and every *moist* situation contain much of it. I am surprised to hear any gentlemen asserting that *simple moisture* is not unwholesome; every unprejudiced medical man knows the contrary; knows that it is as injurious as almost any common kind of bad air whatever. I must however observe, in excuse for these gentlemen, that air is not moist accordingly as it *appears* to be so; for hot air will suspend a very large quantity of moisture without rendering it visible; and cold air will precipitate, and render visible a very small quantity; hence the West Indies both rust metals very quickly, and are (partly from moisture,) unhealthy, though the air is clear; and hence people on the banks of Newfoundland, are not often very unhealthy, though they live almost continually in a mist. And as moisture is unhealthy, so clayey soils which do not readily absorb it, are found much less wholesome than gravelly or chalky.—I have, indeed, been informed, intermittents sometimes appear about Newcastle, which can be attributed to nothing else but the evaporation from a clayey soil. It is not to man alone, but to other animals that moisture is pernicious; sheep, for instance, readily take the rot by the evaporation of the water from a clayey soil; but more readily, certainly, if there be any thing putrid with the water, as rotten turnips. Scotland, according to my judgment, is not a moist climate, but in general a dry one; and it remains free from intermittents, (according to my opinion,) and healthy, chiefly from its being so; for though mists hang often on the mountains and sometimes descend, they do not in general remain down long, and the visibility of vapour is neither a proof of the quantity of it, nor of its fitness for passing the throat into the lungs.—We have, indeed, a good reason why Scotland should not in general be very moist; I have scarcely seen any *strong clay* in it; the soil in general seems a brown mould, which is very well qualified for *absorbing* wet: and, indeed, in the conversations which I have had with the most skilful agriculturists, who know the modes of culture generally used both in England and Scotland, I find that they exultingly notice that the Scotch *always* plow only with *two horses;* thinking that they have from this greatly surpassed the English in agriculture:—but they *could not* do this, had they a strong clay soil to deal with; especially with their own horses, which are very weak and small, compared with the English; for a layey soil may be seen in many places in England, so hat three or four strong horses can only just draw the plow along: the ploughs too, in this part of the island, (though extremely proper for the soil, and ought to be adopted in an easily managed soil in England,) are so light and weak, that a strong clay soil would soon break them to pieces; all which circumstances, I think, prove that there is but very little strong clay in Scotland.—I think upon the whole, therefore, that Scotland has *in general* a dry air, and is, in some measure, on that account, free from intermittents; and also from other fevers, except in particular situations. In crowded streets, and in the west of Scotland, which is in many places extremely moist, bad fevers are not uncommon. A gentleman who has practised at Paisley, has told me, that in the crowded parts of that town, (which too are very moist,) a bad fever is very common; and has several times been attended with buboes.

All crowded, dirty towns, are sources of an endemic bad air; also a long calm over such places, or any other, where much bad air is generated, is particularly noxious, as it lets the bad air accumulate there; indeed, it is said that Vienna had even the plague from a stagnation of the air for three months, in hot weather.

There is another general cause too of that bad air, which may may be called endemic: it is cold succeeding great heat. The heat raises much moisture, &c. and the cold succeeding, makes it fall down again near the earth. This is the reason why the air in hot fine weather, is often unhealthy about sun-set; and also one reason why the latter end of the year is so foggy and unhealthy in England.

Many other sources of endemic bad air might be particularized; but most of them may present themselves, by reflecting on those already mentioned; and on the epidemic and sporadic instances of bad air. It should not, however, be forgotten, that in some places, particularly in volcanic countries, an endemic bad air arises from an epidemic source; that is, from terrestrial expirations, either from the mouths of the volcanos, from grottos, or from sudden openings in the earth.

*Sporadic bad air.*—It is surprising, how much we are exposed to what I may call a *sporadic* bad air; and how little care we take to guard against it. And I notice this, the rather as I am persuaded sporadic bad air is very fatal to numbers of all ages; and that by its being avoided, an endemic or epidemic bad air would, in general, have much less effect; though, in noticing this kind of bad air, I must dwell upon very vulgar, and therefore what may *seem* very *unphilosophical* circumstances. The sporadic bad air injures us more or less from infancy to old age, and in general more than either the endemic or the epidemic; but in some of us more than others. Children are much exposed to it; they often get turned on one side upon the pillow, or get the bed-clothes over their nose, so that they cannot easily breathe; and often get a handkerchief or apron thrown *purposely* over their face, to make them *go to sleep.* I have frequently seen this; and as it induces an apoplectic tendency, by impeding the discharge of blood from the veins, it is highly injurious, although it may *often* succeed in making a child sleep. Young people too, and adults as well as infants, suffer from want of proper air, especially in the night: some have the custom of sleeping almost on their faces, (as long as they can do this) *from choice.* A boy was brought to me about 10 years old, emaciated and dejected to a surprising degree, and yet his mother said she could not tell why he became so; and that half a year before, he was a fine healthy, active boy: and besides emaciation and dejection, he had a pain in his abdomen, coming on in the night, and at the same time, violent fits. I asked him and his mother, how (that is, in what manner) *he slept?* and found he slept in a rather unventilated chamber, ber, and had the custom of *lying on his face*. I frightened him from doing that in future, and requested the mother would get him as airy a bed as she could; in a few months the child became quite well again, and went thro' the natural small-pox very well, and remained in good health; though for the former complaints he took no medicines.

Adults often sleep with the curtains drawn all around the bed; or they sleep two together in a very small bed, with curtains drawn round, or a wall on two sides, and consequently, often breathing each others breath, or their own again; or they sleep in rooms without fire-places, or without large windows, or even in bed closets, which are the worst of all. And it is only, I believe, in the higher circles where most of these sporadic sources of bad air do not much act; (they are exceedingly powerful in the lower classes.) But to counterbalance the exemption which the higher classes have from bad air from these sources, they are often equally as much exposed to it from others; for instance, oratorios, concerts, assemblies, plays, routs, and even some churches, are extremely full of bad air; and coffee-houses very much abound with it; but (which, however, concerns only a few in the middle orders of society, and these not often) stage coaches are sometimes almost destructive from it. In a long journey performed *all at once*, as from London to Edinburgh, many have *died* from bad air from this cause, generally apoplectic.—For these reasons, I, who am particularly susceptible of injury from bad air, have made it a point to go but little to any of these places for several years; and when I do go, to stay as short a time as possible. Private close carriages too, (though used, as it is said, to take *an airing*,) when crouded, are by no means healthy. And it is remarkable, that people who use a dining room and drawing room, do not always have the empty room well aired before they go into it again; and that some people who live constantly in one room, seldom have the window opened for days together. We have reason then to conclude, that sporadic bad air is very much applied, as well as epidemic and endemic.

I have particularized all these sources of bad air for many reasons; but chiefly, in order to bring the attention of medical gentlemen to be fixed more upon the nature and the quantity of the air we breathe; and also to shew the mode of operation of some of the most general causes of diseases.

*Of*

*Of the proximate causes of diseases.*—I think it will appear evident to every unprejudiced reflecting mind, that these causes of disease can act upon the body essentially, only through the medium of the lungs; and I could wish it to seem as clear, that they act principally *upon the lungs instantaneously without absorption:* for the doctrines of the oxygenation, and de-oxygenation of the system, are not true, and have never been taught by me, though they have often been attributed to me, and often too with much respect.

I maintain that the chief effect vital air has upon the system, is by stimulating the vessels in the lungs, *the moment it is applied to them;* and that though part of that so inspired, is taken into the blood-vessels, when it has entered into the blood-vessels its principal action is *over,* it having then ceased to be in a *free state,* in which *alone* it acts *as vital air.*

All the humours of the body may be oxygenated, or de-oxygenated by the kind of food used, without material or any deviation from health; and, indeed, oxygenous, or hydrogenous, or azotic kinds of food, may all be used to very considerable extent, without danger or injury.—But the breathing of oxygen gas, or hydrogen gas, or azotic gas, *has almost sudden and amazing effect upon the system,* and clearly before it is absorbed and carried into the whole circulation.

My doctrines, therefore, are all founded on the *instantaneous effect* which the gases have upon the *lungs.*

I never, in any of my publications, admitted the doctrines of the oxygenation of the system, though I always treated of the effects of oxygen, and of the other gases. I beg to notice this now most seriously, because I have been much charged with a dereliction of doctrines, of which I was supposed among the original authors, and for which I have received some compliments; and also of being guilty of constant and great *contradictions:* but I trust gentlemen who reflect on this subject must see, that I can treat of the effects of oxygen without saying a word about its absorption, or noticing whether it be in a large or in a small quantity in the blood.

As this circumstance, however, (for it to have full force) requires many wrongly associated ideas and prejudices to be laid aside, and then that it should be calmly reflected upon for a considerable time, I shall not pursue the reflections on the proximate causes of diseases, till I am persuaded gentlemen *in general* can attend to the operations of the the gases, and other similar agents, (among which may be reckoned *deprivation of air)* without alluding to the *oxygenation* or *de-oxygenation* of the SYSTEM.

I am, &c.

THOMAS ALDER.

*Edinburgh, February* 6, 1808.

( To be continued. )

